# A Crucial Role for the Small GTPase Rac1 Downstream of the Protein Kinase Akt2 in Insulin Signaling that Regulates Glucose Uptake in Mouse Adipocytes

**DOI:** 10.3390/ijms20215443

**Published:** 2019-10-31

**Authors:** Nobuyuki Takenaka, Mika Nakao, Sayaka Matsui, Takaya Satoh

**Affiliations:** Laboratory of Cell Biology, Department of Biological Science, Graduate School of Science, Osaka Prefecture University, Sakai, Osaka 599-8531, Japan; takenaka@b.s.osakafu-u.ac.jp (N.T.); nakao17@b.s.osakafu-u.ac.jp (M.N.); round.syk0816@gmail.com (S.M.)

**Keywords:** adipocyte, Akt2, glucose uptake, GLUT4, GTPase, insulin, Rac1

## Abstract

Insulin-stimulated glucose uptake is mediated by translocation of the glucose transporter GLUT4 to the plasma membrane in adipocytes and skeletal muscle cells. In both types of cells, phosphoinositide 3-kinase and the protein kinase Akt2 have been implicated as critical regulators. In skeletal muscle, the small GTPase Rac1 plays an important role downstream of Akt2 in the regulation of insulin-stimulated glucose uptake. However, the role for Rac1 in adipocytes remains controversial. Here, we show that Rac1 is required for insulin-dependent GLUT4 translocation also in adipocytes. A Rac1-specific inhibitor almost completely suppressed GLUT4 translocation induced by insulin or a constitutively activated mutant of phosphoinositide 3-kinase or Akt2. Constitutively activated Rac1 also enhanced GLUT4 translocation. Insulin-induced, but not constitutively activated Rac1-induced, GLUT4 translocation was abrogated by inhibition of phosphoinositide 3-kinase or Akt2. On the other hand, constitutively activated Akt2 caused Rac1 activation, and insulin-induced Rac1 activation was suppressed by an Akt2-specific inhibitor. Moreover, GLUT4 translocation induced by a constitutively activated mutant of Akt2 or Rac1 was diminished by knockdown of another small GTPase RalA. RalA was activated by a constitutively activated mutant of Akt2 or Rac1, and insulin-induced RalA activation was suppressed by an Akt2- or Rac1-specific inhibitor. Collectively, these results suggest that Rac1 plays an important role in the regulation of insulin-dependent GLUT4 translocation downstream of Akt2, leading to RalA activation in adipocytes.

## 1. Introduction

Insulin stimulates the uptake of blood glucose in skeletal muscle and adipose tissue, which depends on the translocation of the glucose transporter GLUT4 to the plasma membrane [1,2,3]. GLUT4 is sequestered in specific intracellular compartments termed GLUT4 storage vesicles in unstimulated cells. Following insulin stimulation, GLUT4-containing vesicles are translocated and fused to the plasma membrane, and consequently GLUT4 molecules that mediate facilitated diffusion of circulating glucose into the cell across the plasma membrane are significantly increased.

Signaling mechanisms underlying insulin-stimulated translocation of GLUT4 from intracellular storage sites to the plasma membrane have been intensively studied over the past few decades [1,2,3,4,5]. Various signaling molecules function downstream of the activated insulin receptor, leading to the enhancement of GLUT4 vesicle transport to the plasma membrane. Particularly, phosphoinositide 3-kinase (PI3K) and the protein kinase Akt2 serve as key elements of this signal transduction in both skeletal muscle cells and adipocytes. Phosphorylation of a variety of substrate proteins by Akt2 is considered to be crucial for further downstream signaling events. In addition to the signaling pathway that is common between these two types of cells, an adipocyte-specific Akt2-independent pathway involving the Cbl proto-oncogene product, CAP and CrkII adaptor proteins, the guanine nucleotide exchange factor (GEF) C3G, and the Rho family small GTPase TC10 has been identified [6,7].

Recently, we and others have identified the Rho family small GTPase Rac1 as a critical component of insulin-dependent signaling for glucose uptake in skeletal muscle [4,5,8,9,10,11,12,13]. A pivotal role for Rac1 in insulin-stimulated glucose uptake was originally revealed in cultured myoblasts and myotubes [8,9,10,12]. Shortly thereafter, the involvement of Rac1 in insulin signaling was further confirmed by the use of mouse skeletal muscle [11,13]. Moreover, impaired glucose tolerance and higher plasma insulin concentrations after intraperitoneal glucose injection were observed in muscle-specific *rac1* knockout mice, demonstrating that Rac1 actually plays a physiologically important role in insulin action in skeletal muscle [11].

The involvement of Rac1 in insulin-stimulated glucose uptake in adipocytes was not supported by the observation that neither constitutively activated nor dominant-negative Rac1 mutants, when ectopically expressed, affected glucose uptake in 3T3-L1 adipocytes in a previous study [14]. In contrast, the GEF P-Rex1 has been identified as a regulator of PI3K-dependent GLUT4 translocation in response to insulin in 3T3-L1 adipocytes, suggesting that Rac1 may be implicated in adipocyte insulin signaling [15]. In fact, P-Rex1-facilitated GLUT4 trafficking occurred in a Rac1-dependent manner [15]. Therefore, the involvement of Rac1 in adipocytes remains controversial.

In addition to Rac1, another small GTPase RalA, which belongs to the Ras family, has been implicated as a switch for insulin signaling in adipocytes [16,17]. RalA is activated in response to insulin in 3T3-L1 adipocytes and mouse white adipocytes [16,17]. Activated RalA, which is localized in GLUT4-containing vesicles, binds to the exocyst complex, and thereby tethers GLUT4 vesicles to the plasma membrane [16]. The activation of RalA was also detected in cultured myoblasts and mouse skeletal muscle following insulin stimulation in vitro and in vivo, respectively [18,19]. Particularly, RalA activation in response to insulin occurred in a Rac1-dependent manner [19]. Furthermore, insulin-dependent GLUT4 translocation was inhibited when RalA was knocked down in mouse skeletal muscle fibers [19]. Therefore, it is likely that RalA is also involved in skeletal muscle insulin signaling, and serves as a regulator of glucose uptake downstream of Rac1.

In this study, we tested whether Rac1 participates in insulin-dependent signaling that regulates glucose uptake in adipocytes. We provide evidence that Rac1 indeed acts as a regulator of GLUT4 translocation downstream of Akt2. Moreover, we show that Rac1 regulates GLUT4 translocation in a RalA-dependent manner.

## 2. Results

### 2.1. Establishment of the L1-GLUT4 Cell Line and Its Differentiation to Adipocytes In Vitro

**Figure 1:**
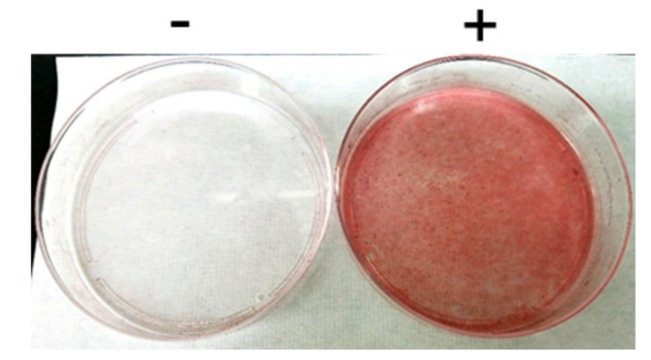
Oil Red O staining of L1-GLUT4 cells. L1-GLUT4 cells before (-) and after (+) the induction of in vitro differentiation for 14 days were subjected to Oil Red O staining.

A GLUT4 reporter containing green fluorescent protein (GFP) and exofacial Myc tags (GLUT4*myc*7-GFP) [13,19,20,21,22,23] was employed to assess the translocation of GLUT4 to the plasma membrane. A 3T3-L1-derived cell line that stably expresses GLUT4*myc*7-GFP, designated L1-GLUT4, was established and used in this study. Differentiation of L1-GLUT4 fibroblasts to adipocytes was induced as described in Materials and Methods, and differentiation to adipocytes was monitored by Oil Red O staining (Figure 1). Thereafter, in vitro-differentiated L1-GLUT4 adipocytes that contain cytoplasmic lipid droplets were isolated via density gradient centrifugation and subjected to subsequent analyses.

### 2.2. Effect of a Rac1-Specific Inhibitor on GLUT4 Translocation Induced by Insulin or a Constitutively Activated Mutant of PI3K or Akt2

**Figure 2:**
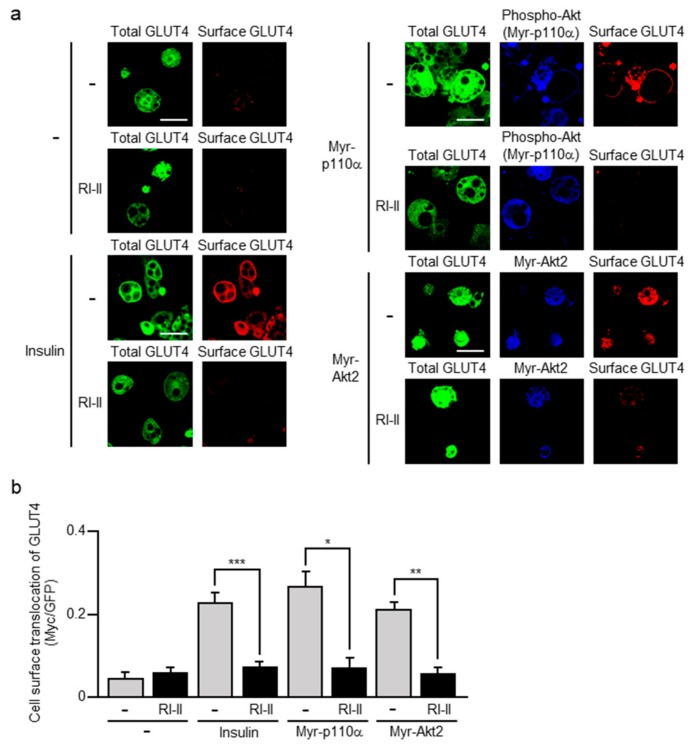
Effect of RI-II on GLUT4 translocation induced by insulin or a constitutively activated mutant of phosphoinositide 3-kinase (PI3K) or Akt2. (**a**) L1-GLUT4 adipocytes were infected with the control (-), an N-terminally myristoylated form of the phosphoinositide 3-kinase catalytic subunit p110α (Myr-p110α)-expressing, or an N-terminally myristoylated form of Akt2 (Myr-Akt2)-expressing virus. The total amount of GLUT4*myc*7-green fluorescent protein (GFP) was estimated by the fluorescence of GFP. The expression of Myr-p110α was monitored by immunofluorescent microscopy using an anti-phospho-Akt antibody. Myr-Akt2 was visualized by immunofluorescent microscopy using an anti-hemagglutinin (HA) antibody. Cell surface-localized GLUT4*myc*7-GFP was visualized by immunofluorescent microscopy using an anti-Myc antibody. Scale bar, 50 μm. (**b**) Cell surface translocation of GLUT4*myc*7-GFP shown in (**a**) was quantified. Data are shown as means ± S.E. (*n* = 10). * *p* < 0.05; ** *p* < 0.01; *** *p* < 0.001.

Following insulin stimulation, the GLUT4 reporter translocated to the plasma membrane was indeed detected by an anti-Myc antibody (Figure 2). The lipid kinase PI3K and the protein kinase Akt2 are known to play a pivotal role in insulin-stimulated glucose uptake in adipocytes. Therefore, we tested whether constitutively activated forms of these kinases enhance GLUT4 translocation. An N-terminally myristoylated form of the phosphoinositide 3-kinase catalytic subunit p110α (Myr-p110α) and an N-terminally myristoylated form of Akt2 (Myr-Akt2), which are known as constitutively activated mutants, were ectopically expressed, and GLUT4 translocation was examined. In this study, we used a Myr-p110α construct without an epitope tag, and therefore the expression of Myr-p110α was monitored by immunofluorescent microscopy for the phosphorylated form of Akt (phospho-Akt) as described previously [17]. Phosphorylation of Akt is regulated downstream of PI3K, and actually phospho-Akt became detectable in response to the expression of Myr-p110α whereas it was not detected in unstimulated 3T3-L1 adipocytes. Myr-Akt2 was expressed as a hemagglutinin (HA)-tagged form, and detected by immunofluorescent microscopy for the HA tag. As expected, both Myr-p110α and Myr-Akt2 induced GLUT4 translocation (Figure 2). Interestingly, prior treatment of L1-GLUT4 adipocytes with the Rac1-specific inhibitor RI-II almost completely suppressed the translocation of GLUT4 to the plasma membrane (Figure 2). These results support the idea that Rac1 is involved in insulin-dependent GLUT4 translocation, acting downstream of Akt2.

### 2.3. Effect of PI3K- and Akt2-Specific Inhibitors on GLUT4 Translocation Induced by a Constitutively Activated Mutant of Rac1

**Figure 3:**
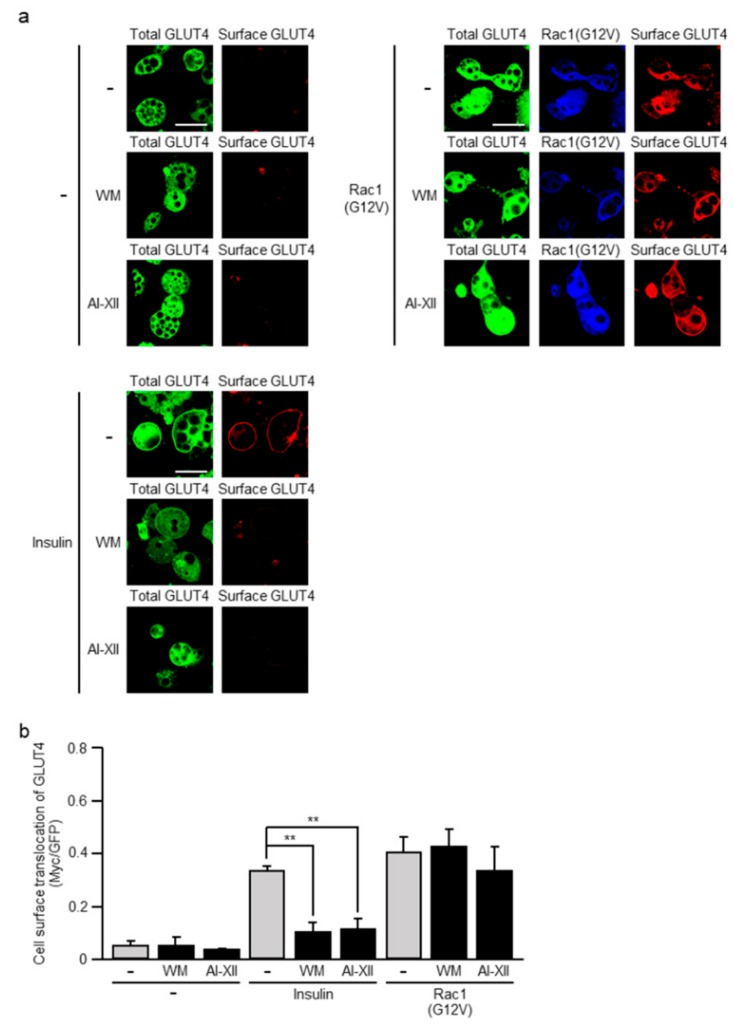
Effect of wortmannin (WM) and AI-XII on GLUT4 translocation induced by a constitutively activated mutant of Rac1. (**a**) L1-GLUT4 adipocytes were infected with the control (-) or Rac1(G12V)-expressing virus. The total amount of GLUT4*myc*7-GFP was estimated by the fluorescence of GFP. Rac1(G12V) was visualized by immunofluorescent microscopy using an anti-HA antibody. Cell surface-localized GLUT4*myc*7-GFP was visualized by immunofluorescent microscopy using an anti-Myc antibody. Scale bar, 50 μm. (**b**) Cell surface translocation of GLUT4*myc*7-GFP shown in (a) was quantified. Data are shown as means ± S.E. (*n* = 10). ** *p* < 0.01.

We previously demonstrated that, in L6 myoblasts, ectopic expression of a constitutively activated mutant of Rac1 induced GLUT4 translocation, suggesting that Rac1 is involved in insulin-dependent glucose uptake [12]. Therefore, we next examined whether a constitutively activated mutant of Rac1, when ectopically expressed, induces GLUT4 translocation in L1-GLUT4 adipocytes. The constitutively activated mutant Rac1(G12V) actually induced GLUT4 translocation when ectopically expressed (Figure 3). Rac1(G12V) was expressed as an HA-tagged form, and detected by immunofluorescent microscopy for the HA tag. We next tested whether PI3K and Akt2 are involved in Rac1(G12V)-induced GLUT4 translocation. For this, we employed specific inhibitors for PI3K and Akt2. In fact, both the PI3K-specific inhibitor wortmannin (WM) and the Akt2-specific inhibitor AI-XII diminished insulin-dependent GLUT4 translocation (Figure 3). In marked contrast, Rac1(G12V)-induced GLUT4 translocation was not suppressed by treatment with WM (Figure 3). AI-XII slightly impaired Rac1(G12V)-induced GLUT4 translocation, but this effect was not statistically significant (Figure 3). These results suggest that neither PI3K nor Akt2 serves as a regulator of GLUT4 translocation downstream of Rac1.

### 2.4. Glucose Uptake Induced by Insulin or a Constitutively Activated Mutant of PI3K, Akt2, or Rac1

**Figure 4:**
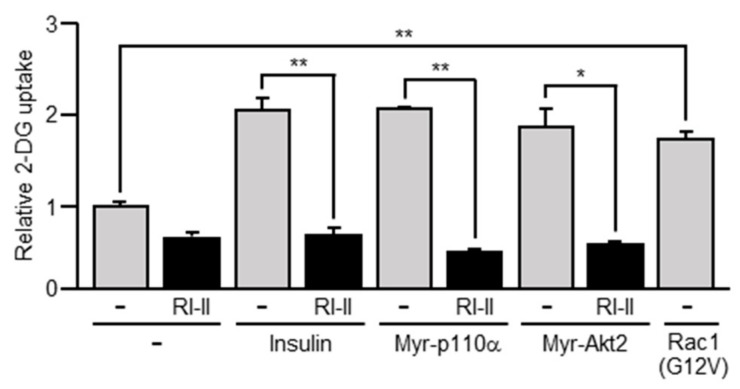
The uptake of 2-deoxy-D-glucose (2-DG) induced by insulin or a constitutively activated mutant of PI3K, Akt2, or Rac1. 3T3-L1 adipocytes were infected with the control (-), Myr-p110α-expressing, Myr-Akt2-expressing, or Rac1(G12V)-expressing virus. The uptake of 2-DG in these adipocytes was measured. The effect of RI-II on the uptake of 2-DG was also tested. Data are shown as means ± S.E. (*n* = 3). * *p* < 0.05; ** *p* < 0.01.

The effect of ectopic expression of a constitutively activated mutant of PI3K, Akt2, or Rac1 on the uptake of 2-deoxy-D-glucose (2-DG) was also examined in in vitro-differentiated 3T3-L1 adipocytes (Figure 4). All of the above constitutively activated mutants as well as insulin in fact enhanced the uptake of 2-DG in 3T3-L1 adipocytes, consistent with the observation that GLUT4 translocation was induced by these mutants. In addition, the Rac1-specific inhibitor RI-II almost completely suppressed the uptake of 2-DG stimulated by insulin or a constitutively activated mutant of PI3K or Akt2 (Figure 4). These results are consistent with the effect of RI-II on GLUT4 translocation.

### 2.5. Involvement of Akt2 in Insulin-Stimulated Activation of Rac1

**Figure 5:**
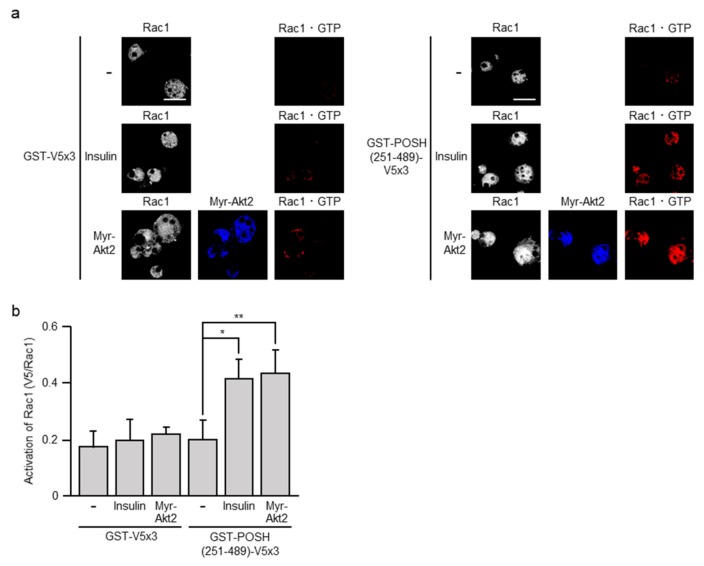
The formation of GTP-bound active Rac1 (Rac1·GTP) by ectopic expression of constitutively activated Akt2. (**a**) 3T3-L1 adipocytes were infected with the control (-) or Myr-Akt2-expressing virus. Endogenous Rac1 was visualized by immunofluorescent microscopy using an anti-Rac1 antibody. Myr-Akt2 was visualized by immunofluorescent microscopy using an anti-HA antibody. Rac1·GTP was visualized by immunofluorescent microscopy using an anti-V5 antibody after treatment with glutathione *S*-transferase (GST)-POSH(251–489)-V5×3, but not GST-V5×3. Scale bar, 50 μm. (**b**) The formation of Rac1·GTP shown in (a) was quantified. Data are shown as means ± S.E. (*n* = 10). * *p* < 0.05; ** *p* < 0.01.

We recently detected the formation of GTP-bound active Rac1 (Rac1·GTP) in 3T3-L1 adipocytes following insulin stimulation, suggesting a role for Rac1 in insulin signaling [17]. Here, we examined the effect of ectopic expression of constitutively activated Akt2 on the activation state of Rac1. A polypeptide that specifically recognizes Rac1·GTP, designated glutathione *S*-transferase (GST)-POSH(251–489)-V5×3 [12,20,21,22,24,25], was used as a probe. When ectopically expressed, Myr-Akt2 actually induced the formation of Rac1·GTP as detected by the binding of GST-POSH(251–489)-V5×3, but not the control peptide GST-V5×3 (Figure 5). Therefore, it is possible that Akt2 may act as an upstream regulator of Rac1 in insulin signaling in adipocytes as in skeletal muscle.

**Figure 6:**
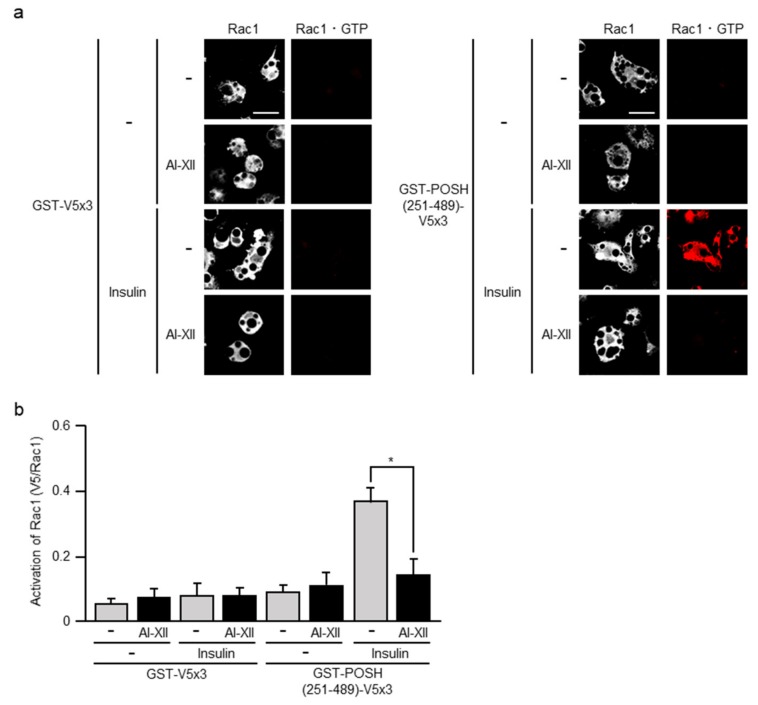
Effect of AI-XII on insulin-induced formation of Rac1·GTP. (**a**) Endogenous Rac1 was visualized by immunofluorescent microscopy using an anti-Rac1 antibody. Rac1·GTP was visualized by immunofluorescent microscopy using an anti-V5 antibody after treatment with GST-POSH(251–489)-V5×3, but not GST-V5×3. Scale bar, 50 μm. (**b**) The formation of Rac1·GTP shown in (a) was quantified. Data are shown as means ± S.E. (*n* = 10). * *p* < 0.05.

To further explore the above possibility, we next examined the effect of AI-XII. Actually, the treatment of 3T3-L1 adipocytes with AI-XII prior to insulin stimulation significantly reduced the formation of Rac1·GTP (Figure 6). Thus, Akt2 may mediate the insulin-triggered signal that controls Rac1 also in adipocytes.

### 2.6. Effect of RalA Knockdown on GLUT4 Translocation Induced by Insulin or a Constitutively Activated Mutant of PI3K, Akt2, or Rac1

RalA belongs to the Ras family of small GTPases and has been characterized as a regulator of GLUT4 translocation in adipocytes and skeletal muscle [16,17,18,19]. RalA acts downstream of Rac1 in skeletal muscle [18,19], but it remains unclear whether this is true in adipocytes as well. To test the possibility that RalA may also act downstream of Rac1 in adipocytes, we next knocked down the expression of RalA, and then examined its effect on GLUT4 translocation. Small hairpin RNA (shRNA) for RalA was introduced into L1-GLUT4 cells by lentivirus-mediated gene transfer, and thereafter puromycin-resistant cells were collected. Differentiation of puromycin-resistant cells to adipocytes was induced as described in Materials and Methods. Knockdown of RalA in differentiated puromycin-resistant adipocytes was confirmed by immunofluorescent microscopy.

**Figure 7:**
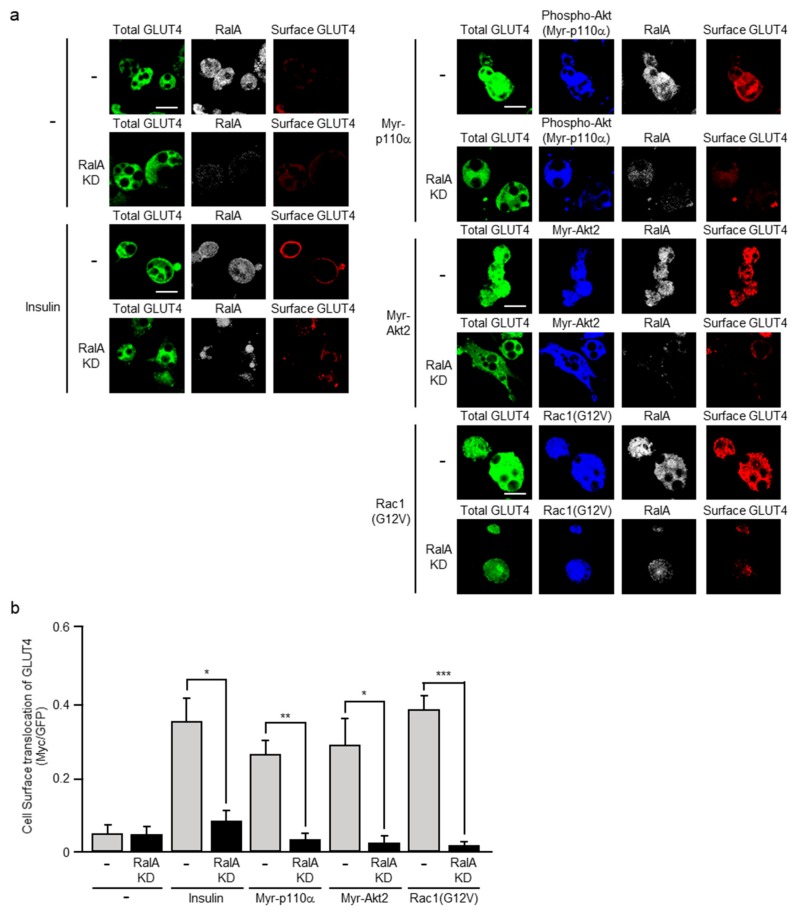
Effect of RalA knockdown on GLUT4 translocation induced by insulin or a constitutively activated mutant of PI3K, Akt2, or Rac1. (**a**) L1-GLUT4 adipocytes were infected with the control (-), Myr-p110α-expressing, Myr-Akt2-expressing, or Rac1(G12V)-expressing virus. The total amount of GLUT4*myc*7-GFP was estimated by the fluorescence of GFP. The expression of Myr-p110α was monitored by immunofluorescent microscopy using an anti-phospho-Akt antibody. Myr-Akt2 and Rac1(G12V) were visualized by immunofluorescent microscopy using an anti-HA antibody. Endogenous RalA was visualized by immunofluorescent microscopy using an anti-RalA antibody. Cell surface-localized GLUT4*myc*7-GFP was visualized by immunofluorescent microscopy using an anti-Myc antibody. Scale bar, 50 μm. (**b**) Cell surface translocation of GLUT4*myc*7-GFP shown in (a) was quantified. Data are shown as means ± S.E. (*n* = 10). * *p* < 0.05; ** *p* < 0.01; *** *p* < 0.001.

Constitutively activated mutants of PI3K, Akt2, and Rac1 were ectopically expressed in the above RalA-knockdown and control L1-GLUT4 adipocytes, and GLUT4 translocation was visualized (Figure 7). Constitutively activated PI3K, Akt2, and Rac1 as well as insulin induced GLUT4 translocation in control L1-GLUT4 adipocytes as expected. In marked contrast, neither insulin nor the above constitutively activated mutants significantly induced GLUT4 translocation in RalA-knockdown L1-GLUT4 adipocytes. These results support the notion that RalA indeed regulates insulin-stimulated translocation of GLUT4 downstream of not only PI3K and Akt2, but also Rac1 in adipocytes.

### 2.7. Involvement of Akt2 and Rac1 in Insulin-Stimulated Activation of RalA

**Figure 8:**
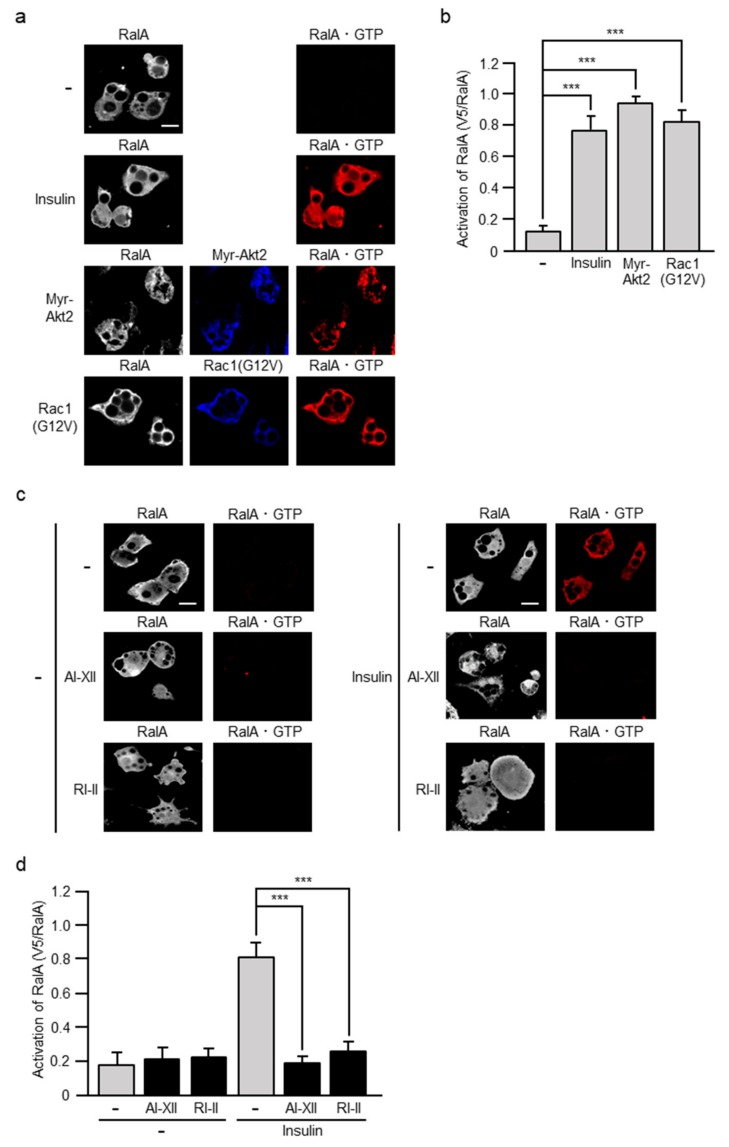
Involvement of Akt2 and Rac1 in insulin-stimulated activation of RalA. (**a**) 3T3-L1 adipocytes were infected with the control (-), Myr-Akt2-expressing, or Rac1(G12V)-expressing virus. Endogenous RalA was visualized by immunofluorescent microscopy using an anti-RalA antibody. Myr-Akt2 and Rac1(G12V) were visualized by immunofluorescent microscopy using an anti-HA antibody. RalA·GTP was visualized by immunofluorescent microscopy using an anti-V5 antibody after treatment with GST-V5×3-Sec5(1–99). Scale bar, 50 μm. (**b**) The formation of Rac1·GTP shown in (a) was quantified. Data are shown as means ± S.E. (*n* = 15). *** *p* < 0.001. (**c**) Effect of AI-XII and RI-II on insulin-induced formation of RalA·GTP. Endogenous RalA was visualized by immunofluorescent microscopy using an anti-RalA antibody. RalA·GTP was visualized by immunofluorescent microscopy using an anti-V5 antibody after treatment with GST-V5×3-Sec5(1–99). Scale bar, 50 μm. (**d**) The formation of RalA·GTP shown in (c) was quantified. Data are shown as means ± S.E. (*n* = 15). *** *p* < 0.001.

The activation of RalA can be assessed by measuring the increase in the RalA·GTP level as in the case of Rac1. We recently detected RalA·GTP by the specific polypeptide probe GST-V5×3-Sec5(1–99) in adipocytes, demonstrating that PI3K is involved in insulin-dependent activation of RalA [17]. Considering that Akt2 and Rac1 may be involved in the regulation of RalA also in adipocyte insulin signaling as described above, we next tested whether RalA is activated in response to the activation of the Akt2-Rac1 pathway. Ectopic expression of a constitutively activated mutant of Akt2 or Rac1 indeed stimulated the formation of RalA·GTP (Figure 8). Finally, the effect of Akt2-specific and Rac1-specific inhibitors on insulin-dependent activation of RalA was examined. As expected, insulin failed to stimulate the formation of RalA·GTP in 3T3-L1 adipocytes treated with an Akt2- specific or Rac1-specific inhibitor prior to insulin challenge (Figure 8).

## 3. Discussion

In skeletal muscle, Rac1 plays a pivotal role in insulin signaling that governs glucose uptake via the translocation of GLUT4 to the sarcolemma [4,5]. In this study, we provide evidence that Rac1 is also implicated in insulin-stimulated glucose uptake in adipocytes. Particularly, (1) reduction of Akt2-dependent translocation of GLUT4 by the inhibition of Rac1 (Figure 2), (2) Rac1 activation by constitutively activated Akt2 (Figure 5), (3) reduction of insulin-dependent Rac1 activation by the inhibition of Akt2 (Figure 6) strongly support the idea that Rac1 acts downstream of Akt2 as in the case of skeletal muscle cells [5,13,20,21,22,25,26]. Recently, we demonstrated that Rac1 is activated downstream of PI3K following insulin stimulation in 3T3-L1 adipocytes [17]. This result also supports our conclusion in this study. Additionally, the activation of Rac1 downstream of PI3K was demonstrated in primary cultured mouse adipocytes and mouse adipose tissue after in vitro and in vivo insulin stimulations, respectively [17]. Thus, it is important to further confirm the involvement of Akt2 and Rac1 in insulin-dependent glucose uptake in these types of cells, and we are now trying to perform these experiments.

Previously, Marcusohn et al. reported that a dominant-negative Rac1 mutant did not inhibit insulin-stimulated glucose uptake in 3T3-L1 adipocytes, arguing against the involvement of Rac1 downstream of PI3K in insulin signaling of adipocytes [14]. Reasons for the discrepancy between the study by Marcusohn et al. and ours remain unclear, but it is possible that the dominant-negative mutant Rac1(S17N), which was employed in the study by Marcusohn et al., might insufficiently block Rac1 activation, and consequently glucose uptake might not be affected although lamellipodium formation was inhibited [14].

Rho family small GTPases, including Rac1, are directly regulated by approximately 80 GEFs presumably in a manner dependent on the cell type and the signaling pathway. Therefore, the identification of the GEF that is primarily responsible for the activation is important for the understanding of the regulatory mechanisms. We identified the GEF FLJ00068 as a regulator of Rac1 in skeletal muscle insulin signaling [12,20,21,22]. Although the precise mechanisms remain to be clarified, FLJ00068 is translocated to the plasma membrane in response to insulin stimulation, serving as a direct Rac1 regulator downstream of Akt2 [20,21,22]. It remains unknown whether FLJ00068 acts as a Rac1 regulator downstream of Akt2 in adipocytes as well, and now we are studying the possible involvement of this GEF in adipocytes. On the other hand, another Rac1 GEF P-Rex1 has been implicated in insulin-promoted GLUT4 translocation in adipocytes [15]. P-Rex1 is a phosphatidylinositol 3,4,5-trisphosphate-dependent GEF, which enhances cytoskeletal rearrangements and GLUT4 translocation at submaximal insulin concentrations in a Rac1-dependent manner [15]. Therefore, P-Rex1 may be a missing link between PI3K and Rac1 in adipocyte insulin signaling [15]. However, it remains unknown whether Akt2 is required for the activation of P-Rex1, and if not, another unidentified GEF may also be involved in the regulation of Rac1 downstream of Akt2.

Mechanisms whereby Rac1 activates RalA in adipocytes also remain to be clarified. The involvement of RalA in insulin-stimulated GLUT4 translocation was first demonstrated in adipocytes [16], and then in skeletal muscle [18,19]. A Ral GTPase-activating protein (GAP) complex, termed RalGAP2, was reported to act as a RalA regulator in insulin signaling in adipocytes [27]. It has been demonstrated that GAP activity of RalGAP2 is decreased through phosphorylation of its catalytic subunit by Akt2, and consequently RalA is activated [27]. Recently, mice with dramatically increased RalA activity in adipocytes due to adipocyte-specific knockout of the gene for RalGAPB, a scaffolding protein required for stabilization of the RalGAP complex, were generated [28]. These mice indeed exhibited increased glucose disposal in brown adipose tissue, which leads to increased adiposity, lower fasting blood glucose and insulin levels, and improved whole-body glucose handling [28]. Another mechanism in which the GTPase Rab10 induces the activation of RalA through the Ral GEF Rlf/Rgl2 has also been reported in adipocyte insulin signaling leading to glucose uptake [29]. In contrast, a Rac1-dependent regulator of RalA that functions in skeletal muscle insulin signaling has not been identified. In the present study, we provide evidence that RalA activation occurs downstream of Rac1 not only in skeletal muscle, but also in adipocytes (Figure 7 and Figure 8). Therefore, it is possible that an as yet unidentified RalA regulator, either a GEF or a GAP, may act as a link between Rac1 and RalA, and this regulator plays a central role in the regulation of RalA in adipocytes. Further studies will be required to characterize the regulators for Rac1 and RalA in adipocytes.

## 4. Materials and Methods

### 4.1. Materials

A rat monoclonal antibody against the HA epitope tag (11 867 423 001), a mouse monoclonal antibody against the Myc epitope tag (05–724), a rat monoclonal antibody against the Myc epitope tag (GTX10910), and goat polyclonal antibody against the V5 epitope tag (A190–119A) were purchased from Roche Applied Science (Penzberg, Germany), Merck Millipore (Burlington, MA, USA), GeneTex (Irvine, CA, USA) and BETHYL (Montgomery, TX, USA), respectively. Mouse monoclonal antibodies against Rac1 (610650) and RalA (610221) were purchased from BD Biosciences (San Jose, CA, USA). A mouse monoclonal antibody against phospho-(Ser473) Akt (4501) was purchased from Cell Signaling Technology (Danvers, MA, USA). Antibodies against goat IgG, mouse IgG, rabbit IgG, and rat IgG conjugated with CF™ 350/543/647 were purchased from Biotium (Fremont, CA, USA). Insulin was purchased from Eli Lilly (Indianapolis, IN, USA).

### 4.2. Isolation of the L1-GLUT4 Cell Line

The recombinant reterovirus for ectopic expression of the GLUT4 reporter GLUT4*myc*7-GFP was generated as described previously [12]. 3T3-L1 fibroblasts were infected with the GLUT4*myc*7-GFP recombinant reterovirus, and a clone that stably produces GLUT4*myc*7-GFP, termed L1-GLUT4, was isolated.

### 4.3. Cell Culture and In Vitro Differentiation of 3T3-L1 and L1-GLUT4 Cells to Adipocytes

3T3-L1 and L1-GLUT4 fibroblasts were cultivated in Dulbecco’s modified Eagle’s medium (DMEM) (043–30085, WAKO, Osaka, Japan) supplemented with 10% (v/v) fetal bovine serum (Biosera (Kansas City, MO, USA), 100 IU/mL penicillin, and 100 μg/mL streptomycin. In vitro differentiation of 3T3-L1 and L1-GLUT4 fibroblasts to adipocytes was induced essentially as described previously [17]. Briefly, 3T3-L1 and L1-GLUT4 fibroblasts were allowed to grow for 2 days post-confluence, and the culture medium was changed to DMEM supplemented with 10% (v/v) fetal bovine serum, 100 IU/mL penicillin, 100 μg/mL streptomycin, 100 nM insulin, 1 μM dexamethasone, 500 μM 3-isobutyl-1-methylxanthine, and 2 μM rosiglitazone. After 2 days, the culture medium was changed to DMEM supplemented with 10% (v/v) fetal bovine serum, 100 IU/mL penicillin, 100 μg/mL streptomycin, and 100 nM insulin, and 3T3-L1 and L1-GLUT4 cells were further cultivated for 12 days. The culture medium was changed every 2 days. Differentiation to adipocytes was monitored by Oil Red O staining as follows: 3T3-L1 adipocytes were washed twice with phosphate-buffered saline (PBS) and fixed with 100 mg/mL formaldehyde for 10 min. Cells were then washed twice with PBS and once with 60% (v/v) isopropanol, followed by treatment with 1.8 mg/mL Oil Red O (25633–92, Nacalai tesque, Kyoto, Japan) in isopropanol for 20 min. After washing once with 60% (v/v) isopropanol and twice with PBS, Oil Red O staining was assessed by light microscopy (CKX53, Olympus, Tokyo, Japan). Differentiated adipocytes were isolated via Histopaque-1077 (10771, Sigma-Aldrich, St. Louis, MO, USA) density gradient centrifugation, seeded onto plastic plates coated with type I-C collagen (Nitta gelatin, Osaka, Japan), and cultivated in DMEM supplemented with 10% (v/v) fetal bovine serum, 100 IU/mL penicillin, and 100 μg/mL streptomycin.

### 4.4. Detection of GLUT4 Translocation to the Plasma Membrane by a Reporter Assay

The GLUT4 reporter GLUT4*myc*7-GFP was originally described in [23]. Cells were starved in DMEM supplemented with 100 IU/mL penicillin, and 100 μg/mL streptomycin for 3 h prior to stimulation with 100 nM insulin for 30 min. In some experiments, cells were treated with 100 nM WM, 5 μM AI-XII, or 25 μM RI-II during the last 1.5 to 2 h of serum starvation. Cells were fixed with 40 mg/mL paraformaldehyde in PBS for 10 min and were incubated with an anti-Myc tag antibody overnight at 4 °C for the detection of GLUT4*myc*7-GFP translocated to the plasma membrane. After washing three times with PBS, cells were fixed again with 40 mg/mL paraformaldehyde in PBS for 5 min, permeabilized with 0.5% (v/v) Triton X-100 in PBS for 15 min, and incubated in 0.1% (v/v) Triton X-100, 0.05% (v/v) Tween 20, and 0.05% (w/v) sodium azide in PBS supplemented with 2% (v/v) normal goat serum (DAKO, Santa Clara, CA, USA)) for 15 min. Permeabilized cells were further treated with an anti-phospho-Akt (for the identification of cells that carry ectopically expressed Myr-p110α) or anti-HA (for the identification of cells that carry ectopically expressed Myr-Akt2 or Rac1(G12V)) antibody for 2 h. After washing three times with 0.1% (v/v) Tween 20 in PBS or 0.1% (v/v) Tween 20 in Tris-buffered saline, anti-Myc, anti-phospho-Akt, and anti-HA antibodies were detected with fluoresceinated secondary antibodies. Images were obtained and analyzed using a confocal laser-scanning microscope (FV1200, Olympus, Tokyo, Japan). Fluorescent intensities of Myc and GFP in regions of interest were quantified using ImageJ software. The relative amount of GLUT4*myc*7-GFP translocated to the plasma membrane was estimated by the ratio of Myc and GFP fluorescent intensities (Myc/GFP). Values of at least 10 cells in total from six different images for each condition were used for statistical analysis (Student’s *t* test).

### 4.5. Measurement of the Uptake of 2-DG

In vitro-differentiated 3T3-L1 adipocytes were plated at a density of 2 × 10^4^ cells per well in a 96-well cell culture plate. Cells were starved in glucose-free DMEM (A1443001, Thermo Fisher Scientific, Waltham, MA, USA) for 3 h prior to stimulation with 100 nM insulin for 30 min. In some experiments, cells were treated with 25 μM RI-II during the last 2 h of serum starvation. Cells were then incubated with 1 mM 2-DG for 15 min, and the uptake of 2-DG was measured using the Glucose Uptake-Glo assay kit (Promega, Madison, WI, USA)) according to the manufacturer’s instructions. The luminescence signal was measured using a multi-detection microplate reader (Synergy HT, BioTek, Winooski, VT, USA)).

### 4.6. Detection of the Activated Form of Rac1 and RalA

In vitro-differentiated 3T3-L1 adipocytes were starved in DMEM supplemented with 100 IU/mL penicillin, and 100 μg/mL streptomycin for 3 h prior to stimulation with 100 nM insulin for 30 min. In some experiments, cells were treated with 5 μM AI-XII or 25 μM RI-II during the last 1.5 to 2 h of serum starvation. The activated form of Rac1 and RalA was detected essentially as described previously [17]. Briefly, adipocytes were fixed in overlay assay buffer (50 mM HEPES-NaOH (pH 7.3), 150 mM NaCl, 20 mM MgCl2, and 0.05% (v/v) Tween 20) supplemented with 20 mg/mL paraformaldehyde on ice for 1 min. Fixed cells were washed three times with overlay assay buffer and incubated with GST-POSH(251–489)-V5×3 (for the activated form of Rac1, 20 μg/mL) or GST-V5×3-Sec5(1–99) (for the activated form of RalA, 20 μg/mL) in overlay assay buffer supplemented with 0.1% (v/v) Triton X-100 and 50 μg/mL bovine serum albumin on ice for 10 min. GST-POSH(251–489)-V5×3 and GST-V5×3-Sec5(1–99) were purified from *Escherichia coli* transformants as previously described [12]. After washing once with overlay assay buffer, cells were fixed again in overlay assay buffer supplemented with 20 mg/mL paraformaldehyde on ice for 10 min. Fixed cells were washed three times with Tris-buffered saline supplemented with 0.1% (v/v) Tween20, and incubated with an antibody against the V5 tag for the detection of GST-POSH(251–489)-V5×3 or GST-V5×3-Sec5(1–99). Cells were counterstained with an antibody against Rac1 or RalA for the estimation of the total amount of Rac1 or RalA. Ectopically expressed Myr-Akt2 and Rac1(G12V) were detected by an anti-HA tag antibody. Anti-V5, anti-HA, anti-Rac1, and anti-RalA antibodies were detected by fluoresceinated secondary antibodies. Images were obtained and analyzed using a confocal laser-scanning microscope (FV1200, Olympus, Tokyo, Japan). Intensities of fluorescent signals of V5 and Rac1 or RalA in regions of interest were quantified using ImageJ software. The activity of Rac1 or RalA was estimated by the ratio of fluorescence signal intensities (V5/Rac1 or V5/RalA). Values of at least 1510 cells in total from four different images for each condition were used for statistical analysis (Student’s *t* test).

### 4.7. Gene Transfer Into Adipocytes by the Use of Viral Vectors

Recombinant adenoviruses for the ectopic expression of Myr-p110α and triple HA-tagged Rac1(G12V) were previously described [17,26]. 3T3-L1 and L1-GLUT4 adipocytes obtained through the induction of in vitro differentiation and subsequent purification of differentiated lipid-containing cells by density gradient centrifugation were infected with adenoviruses for the expression of Myr-p110α and triple HA-tagged Rac1(G12V). A recombinant adeno-associated virus for the ectopic expression of triple HA-tagged Myr-Akt2 was generated by using the AAVpro helper free system (Takara Bio, Kusatsu, Japan) according to the manufacturer’s instructions. 3T3-L1 and L1-GLUT4 adipocytes obtained through the induction of in vitro differentiation and subsequent purification of differentiated lipid-containing cells by density gradient centrifugation were infected with the adeno-associated virus for the expression of triple HA-tagged Myr-Akt2. A recombinant lentivirus for silencing of RalA was generated by using the MISSION shRNA plasmid DNA vector (Sigma Aldrich, St. Louis, MO, USA) according to the manufacturer’s instructions. L1-GLUT4 adipocytes obtained through the induction of in vitro differentiation and subsequent purification of differentiated lipid-containing cells by density gradient centrifugation were infected with the lentivirus for the expression of a commercially available shRNA sequence for mouse RalA (Sigma Aldrich, St. Louis, MO, USA), and puromycin-resistant cells were collected after cultivation in the culture medium containing 2 μg/mL puromycin for 6 days. Knockdown of RalA in these cells was confirmed by immunofluorescent microscopy.

## Abbreviations

2-DG	2-deoxy-D-glucose
DMEM	Dulbecco’s modified Eagle’s medium
GAP	GTPase-activating protein
GEF	guanine nucleotide exchange factor
GFP	green fluorescent protein
GST	glutathione *S*-transferase
HA	hemagglutinin
Myr-Akt2	an N-terminally myristoylated form of Akt2
Myr-p110α	an N-terminally myristoylated form of the phosphoinositide 3-kinase catalytic subunit p110α
PBS	phosphate-buffered saline
PI3K	phosphoinositide 3-kinase
phospho-Akt	the phosphorylated form of Akt
shRNA	small hairpin RNA
WM	wortmannin

## References

[1] Huang S., Czech M.P. (2007). The GLUT4 glucose transporter. Cell Metab..

[2] Klip A., Pâquet M.R. (1990). Glucose transport and glucose transporters in muscle and their metabolic regulation. Diabetes Care.

[3] Saltiel A.R., Kahn C.R. (2001). Insulin signalling and the regulation of glucose and lipid metabolism. Nature.

[4] Chiu T.T., Jensen T.E., Sylow L., Richter E.A., Klip A. (2011). Rac1 signalling towards GLUT4/glucose uptake in skeletal muscle. Cell. Signal..

[5] Satoh T. (2014). Molecular mechanisms for the regulation of insulin-stimulated glucose uptake by small guanosine triphosphatases in skeletal muscle and adipocytes. Int. J. Mol. Sci..

[6] Chiang S.H., Baumann C.A., Kanzaki M., Thurmond D.C., Watson R.T., Neudauer C.L., Macara I.G., Pessin J.E., Saltiel A.R. (2001). Insulin-stimulated GLUT4 translocation requires the CAP-dependent activation of TC10. Nature.

[7] Kanzaki M., Pessin J.E. (2003). Insulin signaling: GLUT4 vesicles exit via the exocyst. Curr. Biol..

[8] JeBailey L., Rudich A., Huang X., Di Ciano-Oliveira C., Kapus A., Klip A. (2004). Skeletal muscle cells and adipocytes differ in their reliance on TC10 and Rac for insulin-induced actin remodeling. Mol. Endocrinol..

[9] Khayat Z.A., Tong P., Yaworsky K., Bloch R.J., Klip A. (2000). Insulin-induced actin filament remodeling colocalizes actin with phosphatidylinositol 3-kinase and GLUT4 in L6 myotubes. J. Cell Sci..

[10] Randhawa V.K., Ishikura S., Talior-Volodarsky I., Cheng A.W., Patel N., Hartwig J.H., Klip A. (2008). GLUT4 vesicle recruitment and fusion are differentially regulated by Rac, AS160 and RAB8A in muscle cells. J. Biol. Chem..

[11] Sylow L., Jensen T.E., Kleinert M., Højlund K., Kiens B., Wojtaszewski J., Prats C., Schjerling P., Richter E.A. (2013). Rac1 signaling is required for insulin-stimulated glucose uptake and is dysregulated in insulin-resistant murine and human skeletal muscle. Diabetes.

[12] Ueda S., Kataoka T., Satoh T. (2008). Activation of the small GTPase Rac1 by a specific guanine nucleotide exchange factor suffices to induce glucose uptake into skeletal muscle cells. Biol. Cell.

[13] Ueda S., Kitazawa S., Ishida K., Nishikawa Y., Matsui M., Matsumoto H., Aoki T., Nozaki S., Takeda T., Tamori Y. (2010). Crucial role of the small GTPase Rac1 in insulin-stimulated translocation of glucose transporter 4 to the mouse skeletal muscle sarcolemma. FASEB J..

[14] Marcusohn J., Isakoff S.J., Rose E., Symons M., Skolnik E.Y. (1995). The GTP-binding protein Rac does not couple PI 3-kinase to insulin-stimulated glucose transport in adipocytes. Curr. Biol..

[15] Balamatsias D., Kong A.M., Waters J.E., Sriratana A., Gurung R., Bailey C.G., Rasko J.E., Tiganis T., Macaulay S.L., Mitchell C.A. (2011). Identification of P-Rex1 as a novel Rac1-guanine nucleotide exchange factor (GEF) that promotes actin remodeling and GLUT4 protein trafficking in adipocytes. J. Biol. Chem..

[16] Chen X.W., Leto D., Chiang S.H., Wang Q., Saltiel A.R. (2007). Activation of RalA is required for insulin-stimulated Glut4 trafficking to the plasma membrane via the exocyst and the motor protein Myo1c. Dev. Cell..

[17] Takenaka N., Nihata Y., Ueda S., Satoh T. (2017). In situ detection of the activation of Rac1 and RalA small GTPases in mouse adipocytes by immunofluorescent microscopy following in vivo and ex vivo insulin stimulation. Cell. Signal..

[18] Nozaki S., Ueda S., Takenaka N., Kataoka T., Satoh T. (2012). Role of RalA downstream of Rac1 in insulin-dependent glucose uptake in muscle cells. Cell. Signal..

[19] Takenaka N., Sumi Y., Matsuda K., Fujita J., Hosooka T., Noguchi T., Aiba A., Satoh T. (2015). Role for RalA downstream of Rac1 in skeletal muscle insulin signalling. Biochem. J..

[20] Takenaka N., Izawa R., Wu J., Kitagawa K., Nihata Y., Hosooka T., Noguchi T., Ogawa W., Aiba A., Satoh T. (2014). A critical role of the small GTPase Rac1 in Akt2-mediated GLUT4 translocation in mouse skeletal muscle. FEBS J..

[21] Takenaka N., Yasuda N., Nihata Y., Hosooka T., Noguchi T., Aiba A., Satoh T. (2014). Role of the guanine nucleotide exchange factor in Akt2-mediated plasma membrane translocation of GLUT4 in insulin-stimulated skeletal muscle. Cell. Signal..

[22] Takenaka N., Nihata Y., Satoh T. (2016). Rac1 activation caused by membrane translocation of a guanine nucleotide exchange factor in Akt2-mediated insulin signaling in mouse skeletal muscle. PLoS ONE.

[23] Bogan J.S., McKee A.E., Lodish H.F. (2001). Insulin-responsive compartments containing GLUT4 in 3T3-L1 and CHO cells: regulation by amino acid concentrations. Mol. Cell. Biol..

[24] Takenaka N., Nihata Y., Satoh T. (2015). Immunofluorescent detection of the activation of the small GTPase Rac1 in mouse skeletal muscle fibers. Anal. Biochem..

[25] Takenaka N., Araki N., Satoh T. (2019). Involvement of the protein kinase Akt2 in insulin-stimulated Rac1 activation leading to glucose uptake in mouse skeletal muscle. PLoS ONE.

[26] Nozaki S., Takeda T., Kitaura T., Takenaka N., Kataoka T., Satoh T. (2013). Akt2 regulates Rac1 activity in the insulin-dependent signaling pathway leading to GLUT4 translocation to the plasma membrane in skeletal muscle cells. Cell. Signal..

[27] Chen X.W., Leto D., Xiong T., Yu G., Cheng A., Decker S., Saltiel A.R. (2011). A Ral GAP complex links PI 3-kinase/Akt signaling to RalA activation in insulin action. Mol. Biol. Cell.

[28] Skorobogatko Y., Dragan M., Cordon C., Reilly S.M., Hung C.W., Xia W., Zhao P., Wallace M., Lackey D.E., Chen X.W. (2018). RalA controls glucose homeostasis by regulating glucose uptake in brown fat. Proc. Natl. Acad. Sci. USA.

[29] Karunanithi S., Xiong T., Uhm M., Leto D., Sun J., Chen X.W., Saltiel A.R. (2014). (2014) A Rab10:RalA G protein cascade regulates insulin-stimulated glucose uptake in adipocytes. Mol. Biol. Cell.

